# Pre‐Treatment MMP7 Predicts Progressive Idiopathic Pulmonary Fibrosis in Antifibrotic Treated Patients

**DOI:** 10.1111/resp.14894

**Published:** 2025-02-07

**Authors:** Roger M. Li, Dino B. A. Tan, Chantalia Tedja, Wendy A. Cooper, Helen E. Jo, Christopher Grainge, Ian N. Glaspole, Nicole Goh, Samantha Ellis, Peter M. A. Hopkins, Christopher Zappala, Gregory J. Keir, Paul N. Reynolds, Sally Chapman, E. Haydn Walters, Darryl Knight, Svetlana Baltic, HuiJun Chih, Tamera J. Corte, Yuben P. Moodley

**Affiliations:** ^1^ Institute for Respiratory Health Nedlands Western Australia Australia; ^2^ University of Western Australia Crawley Western Australia Australia; ^3^ Centre of Research Excellence in Pulmonary Fibrosis Sydney NSW Australia; ^4^ Royal Prince Alfred Hospital Sydney New South Wales Australia; ^5^ University of Sydney Sydney New South Wales Australia; ^6^ Western Sydney University Parramatta New South Wales Australia; ^7^ John Hunter Hospital Newcastle New South Wales Australia; ^8^ University of Newcastle Callaghan New South Wales Australia; ^9^ The Alfred Hospital Melbourne Victoria Australia; ^10^ Monash University Melbourne Victoria Australia; ^11^ The Austin Hospital Heidelberg Victoria Australia; ^12^ University of Queensland St Lucia Queensland Australia; ^13^ Prince Charles Hospital Queensland Australia; ^14^ University of Adelaide Adelaide South Australia Australia; ^15^ Royal Adelaide Hospital Adelaide South Australia Australia; ^16^ University of Melbourne Melbourne Victoria Australia; ^17^ Royal Hobart Hospital Hobart Tasmania Australia; ^18^ Providence Health Care Research Institute Vancouver British Columbia Canada; ^19^ Curtin University Bentley Western Australia Australia; ^20^ Fiona Stanley Hospital Murdoch Western Australia Australia

**Keywords:** biomarker, disease progression, idiopathic pulmonary fibrosis

## Abstract

**Background and Objective:**

Idiopathic pulmonary fibrosis (IPF) is a chronic progressive lung disease with a poor prognosis. Antifibrotics slow the decline of pulmonary function after 12‐months, but limited studies have examined the role of circulatory biomarkers in antifibrotic treated IPF patients.

**Methods:**

Serum from 98 IPF participants, from the Australian Idiopathic Pulmonary Fibrosis Registry were collected at four time‐points over 1 year post‐antifibrotic treatment and analysed as two separate cohorts. Patients were stratified as progressive, if they experienced ≥ 10% decline in FVC or ≥ 15% decline in DLCO or were deceased within 1 year of treatment initiation: or otherwise as stable. Ten molecules of interest were measured by ELISAs in patient serum.

**Results:**

Baseline MMP7 levels were higher in progressive than stable patients in Cohort 1 (*p* = 0.02) and Cohort 2 (*p* = 0.0002). Baseline MMP7 levels also best differentiated progressive from stable patients (Cohort 1, AUC = 0.74, *p* = 0.02; Cohort 2, AUC = 0.81, *p* = 0.0003). Regression analysis of the combined cohort showed that elevated MMP7 levels predicted 12‐month progression (OR = 1.530, *p* = 0.010) and increased risk of overall mortality (HR = 1.268, *p* = 0.002). LASSO regression identified a multi‐biomarker panel (MMP7, ICAM‐1, CHI3L1, CA125) that differentiated progression more accurately than MMP7 alone. Furthermore, GAP combined with MMP7, ICAM‐1, CCL18 and SP‐D was more predictive of 3‐year mortality than GAP alone.

**Conclusion:**

MMP7 along with a multi‐biomarker and GAP panel can predict IPF progression and mortality, with the potential for optimising management.

## Abstract 2

In idiopathic pulmonary fibrosis (IPF) patients, serum MMP7 levels were higher at baseline (pre‐treatment) and at 1 year of antifibrotic treatment in those with progressive disease, compared to those with stable disease. MMP7 may provide a clinically useful signal for risk of progression and mortality in antifibrotic treated IPF patients.

**See related** editorial

**Summary**

In idiopathic pulmonary fibrosis (IPF) patients, serum MMP7 levels were higher at baseline (pre‐treatment) and at 1 year of antifibrotic treatment in those with progressive disease, compared to those with stable disease. MMP7 may provide a clinically useful signal for risk of progression and mortality in antifibrotic treated IPF patients.

**Abbreviations**
AUC: area under curve
CCL18: C‐C chemokine motif ligand 18
CHI3L1: chitinase‐3‐like protein 1
CXCL13: C‐X‐C chemokine motif ligand 13
DLCO: diffusing capacity for carbon monoxide
ELISA: enzyme linked immunosorbent assay
FGF: fibroblast growth factor
FVC: forced vital capacity
GAP: gender, age, physiology
ICAM‐1: intercellular adhesion molecule 1
IPF: idiopathic pulmonary fibrosis
LASSO: least absolute shrinkage and selection operator
MMP‐7: matrix metalloprotease 7
OPN: osteopontin
PDGF: platelet derived growth factor
%pDLCO: percentage predicted diffusing capacity for carbon monoxide
%pFVC: percentage predicted forced vital capacity
POSTN: periostin
ROC: receiver operating characteristic
SP‐D: surfactant protein D
TGF‐β1: transforming growth factor‐β1
VEGF: vascular endothelial growth factor

## 1 Introduction

Idiopathic pulmonary fibrosis (IPF) is a devastating disease that causes irreversible scarring of the lungs and loss of gas‐exchange units resulting in progressive decline in lung function. The global prevalence is estimated at around 30 per 100,000, but this number is increasing as the population ages [1]. The survival rate is poor with a median of 3–5 years post‐diagnosis in untreated patients [2, 3].

Two antifibrotics, pirfenidone and nintedanib, have been shown to attenuate the rate of decline in IPF [4, 5]. However, antifibrotics incur financial costs both for patients and the healthcare system [6, 7], as well as side effects that impact quality of life and may result in treatment discontinuation [5, 8]. Furthermore, there is heterogeneity in response to antifibrotic agents. Given these issues, circulatory biomarkers that identify patients' likely response to antifibrotic therapy may enhance clinical decision making.

Change in forced vital capacity (FVC), diffusing capacity for carbon monoxide (DLCO) and radiological progression are currently used to monitor treatment response. The major limitation to this approach is that significant lung damage must occur before these changes are detectable [9]. Notably, previous studies have demonstrated that several circulatory molecules are associated with IPF progression [10, 11] and mortality [11, 12], suggesting that circulatory biomarkers may identify a response to antifibrotic therapy before changes in lung function or radiology are evident [13, 14, 15]. We and others have shown prognostic association of these molecules in untreated cohorts. There is limited data on the behaviour of biomarkers in an antifibrotic‐treated cohort [12].

We hypothesize that the circulatory levels of these biomarkers can predict the therapeutic response to antifibrotics in patients with IPF. A selection of 10 biomarkers were measured in the serum of IPF patients pre‐ and up to 12‐months post‐antifibrotic therapy as part of a multi‐centre longitudinal observational study. These biomarkers were selected based on their potential involvement in IPF pathogenesis and have been analysed in a treatment‐naïve cohort from the Australian Idiopathic Pulmonary Fibrosis Registry (AIPFR) [10]. The aim of our current study is to determine if biomarker levels were associated with lung function decline, mortality and response to antifibrotic therapy.

## 2 Methods

### 2.1 Study Cohort

This study utilised serum samples of patients from the AIPFR registry biobank which collects longitudinal patient samples and clinical data. IPF subjects were diagnosed as per a multidisciplinary team meeting at the respective sites and were recruited into the registry from hospitals across Australia; The Alfred (Victoria), Royal Prince Alfred (New South Wales), John Hunter Hospital (New South Wales) and Royal Adelaide Hospital (South Australia). Baseline samples were collected before patients were prescribed antifibrotics and then at three subsequent timepoints at 3‐, 6‐ and 12‐months post‐treatment with parallel FVC and DLCO also recorded. To address the issue of reproducibility of biomarker studies, our study consisted of two cohorts. The first group of patients were recruited from 2017 to 2018 (Cohort 1, *n* = 43) and a second cohort recruited from 2017 to 2020 (Cohort 2, *n* = 55).

To assess progression of disease, patients were classified as progressive (*n* = 41) or stable (*n* = 42) by longitudinal changes in lung function after starting antifibrotic treatment. Patients who experienced a ≥ 10% decline in FVC or ≥ 15% decline in DLCO over 6–12 months post‐treatment were classified as progressive whereas those who did not were classified as stable. Additionally, patients who were deceased within 12 months after their baseline sample were classified as progressive.

Fifteen patients did not have sufficient lung function data to be classified as progressive or stable but were included for overall and 3‐year mortality analysis (*n* = 98). No patients had undergone lung transplantation.

### 2.2 Sample Collection and Processing

Peripheral blood was collected in SST II vacutainer tubes (BD Biosciences, CA, USA), left to clot for ≥ 30 min and centrifuged for separation within 2 h of collection. The separated serum was stored at 4°C overnight prior to temperature‐controlled transport to the Institute for Respiratory Health, then aliquoted and stored at −80°C.

### 2.3 Measurement of Serum Biomarker Levels Using Enzyme Linked Immunosorbent Assay (ELISA)

Serum levels of matrix metalloproteinase‐7 (MMP7), periostin (POSTN), C‐X‐C motif ligand 13 (CXCL13), intercellular adhesion molecule‐1 (ICAM‐1), osteopontin (OPN), C‐C motif ligand 18 (CCL18), surfactant protein‐D (SP‐D), chitinase‐3‐like protein‐1 (CHI3L1) and mucin 16 (CA125) were measured by ELISA using DuoSet ELISA Kits (R&D Systems, MN, USA) according to manufacturer's instruction. Serum samples were diluted in 1% bovine serum albumin/phosphate buffered saline to ensure target molecules were in the optimal range for measurement by kit standards.

### 2.4 Statistical Analysis

Categorical data (e.g., gender, treatment, smoking status) between the progressive and stable patients was evaluated using Chi‐squared analyses. Continuous data was analysed using the non‐parametric Mann Whitney U test to compare progressive and stable patients, or the non‐parametric Wilcoxon signed rank test to compare timepoints. Specificity and sensitivity were analysed by the area under the receiver operating characteristic (ROC) curve (AUC).

The association between baseline biomarkers and GAP score with 12‐month progression and overall mortality were analysed in the combined cohort due to small patient‐to‐variable size. Odds ratios for biomarkers and Gender‐Age‐Physiology (GAP) score with 12‐month progression were calculated using multiple and univariable/simple logistic regression. Overall mortality rate was compared using the Kaplan–Meier survival analysis. Time‐to‐event, or overall, mortality risk of biomarkers and GAP was analysed with multivariable and univariable Cox proportional‐hazard regressions. These tests were performed using GraphPad Prism version 9.5.1 (GraphPad, CA, USA) and R version 4.3.2.

### 2.5 LASSO Regression

Least absolute shrinkage and selection operator (LASSO) regressions were performed on R version 4.3.2. LASSO regression was used to create a model of biomarkers and GAP score to predict progression and overall mortality. Missing clinical data was imputed via classification and regression trees. The model generated a predictive value for each patient by selecting a panel of biomarkers (with and without GAP score) that were associated with progression or overall mortality. The predictive value was tested on the same cohort by comparing predicted patient status with the actual status. Using LASSO, baseline parameters were stratified based on their influence on 12‐month progression or 3‐year mortality, outputting a panel of markers that formed a predictive value between 0 and 1 (Table 1; Supporting Information Figure S3) with 0 representing a stable or alive patient and 1 representing a progressive or deceased patient. GraphPad Prism version 9.5.1 was used to evaluate the AUC of 12‐month progression and 3‐year mortality predictive values.

**TABLE 1:** Association of all baseline clinical parameters and biomarker levels with overall mortality using a multivariable Cox Hazard regression.

Association with overall mortality			
Parameters	Hazard ratio	95% CI	*p* value
Gender	0.698	0.253–2.010	0.492
Age	**1.090**	**1.018–1.176**	**0.018**
%pFVC	**0.965**	**0.939–0.990**	**0.008**
%pDLCO	0.991	0.966–1.019	0.520
MMP7 (ng/mL)	**1.256**	**1.017–1.554**	**0.034**
POSTN (ng/mL)	1.008	0.974–1.041	0.637
ICAM‐1 (ng/mL)	1.378	0.724–2.473	0.301
CXCL13 (pg/mL)	1.000	0.999–1.001	0.617
OPN (ng/mL)	0.996	0.978–1.012	0.626
SP‐D (ng/mL)	0.997	0.985–1.009	0.659
CHI3L1 (ng/mL)	**1.004**	**1.001–1.007**	**0.014**
CCL18 (ng/mL)	1.000	0.999–1.001	0.483
CA125 (pg/mL)	1.000	0.997–1.002	0.729
Harrell's C‐statistic		0.77	

*Note*: Bold values highlight statistically significant results.

Abbreviations: CI, confidence interval; CA125, cancer antigen‐125 (mucin 16); CCL18, C‐C motif ligand 18; CHI3L1, chitinase‐3‐like protein‐1; CXCL13, C‐X‐C motif ligand 13; DLCO, diffusing capacity of the lungs for carbon monoxide; FVC, forced vital capacity; ICAM‐1, intercellular adhesion molecule‐1; MMP7, matrix metalloproteinase‐7; OPN, osteopontin; POSTN, periostin; SP‐D, surfactant protein‐D.

A 3‐year mortality cut off was implemented to reflect the median survival time and gave a sufficient cohort size compared to 1‐year and 2‐year mortality. The deceased cohort consisted of those who passed away ≤ 36 months (*n* = 34) while the alive cohort consisted of those who survived for ≥ 36 months (*n* = 30). The specificity of the predictive values was assessed via AUC of ROC analysis by comparing predicted status with the actual status (Figure 3).

## 3 Results

### 3.1 Patient Demographics

Baseline and demographic data for the progressive and stable groups are summarised in Table 2. The age of the progressive group was higher compared to the stable group (*p* = 0.02). The proportion of females was higher in progressive cohort (*n* = 14/41, 34%) than in the stable cohort (*n* = 6/42, 14%) (*p* = 0.03). Other baseline clinical parameters including percentage predicted FVC (%pFVC), %pDLCO, GAP score, treatment types and smoking status were similar between the progressive and stable groups (Table 2). The overall survival time of progressive patients was lower than the stable group (median survival of 27 vs. 46 months, *p* < 0.0001; Supporting Information Figure S1). Clinical demographics were similar also between the two cohorts (Supporting Information Table S1).

**TABLE 2:** Patient characteristics.

Characteristics	Progressive^a^ (*n* = 41)	Stable (*n* = 42)	*p* value
Age	75 (71–78)	71 (65–76)	**0.02**
Male	27	36	**0.03**
Female	14	6	
Caucasian	35	36	0.96
Non‐Caucasian	6	6	
BMI	29 (26–31)	28 (26–31)	0.97
%predicted FVC baseline	86 (78–101)	88 (75–98)	0.28
%predicted DLCO baseline	50 (42–62)	56 (47–79)	0.21
GAP Score (mean ± SD)	3.51 ± 1.1	3.3 ± 1.3	0.37
Nintedanib	20	18	0.81
Pirfenidone	15	16	
Nintedanib and Pirfenidone^b^	6	8	
Ever smoked	25	26	0.93
Never smoked	15	15	

*Note*: Continuous variables are displayed as median and interquartile range (IQR) unless stated otherwise. Bold values highlight statistically significant results.

Abbreviations: BMI, body mass index; DLCO, diffusing capacity of the lungs for carbon monoxide; FVC, forced vital capacity; GAP, gender, age and physiology; SD, standard deviation.

^a^ Progressive IPF patients were identified as patients who experienced a ≥ 10% decline in FVC or ≥ 15% decline in DLCO over 6–12 months post‐treatment or who were deceased within 12 months after their baseline blood sample.

^b^ Patients started on Nintedanib or Pirfenidone who then switched to the other during follow up.

### 3.2 Serum MMP7 Is a Marker of Progression in Antifibrotic Treated IPF Patients

**FIGURE 1:**
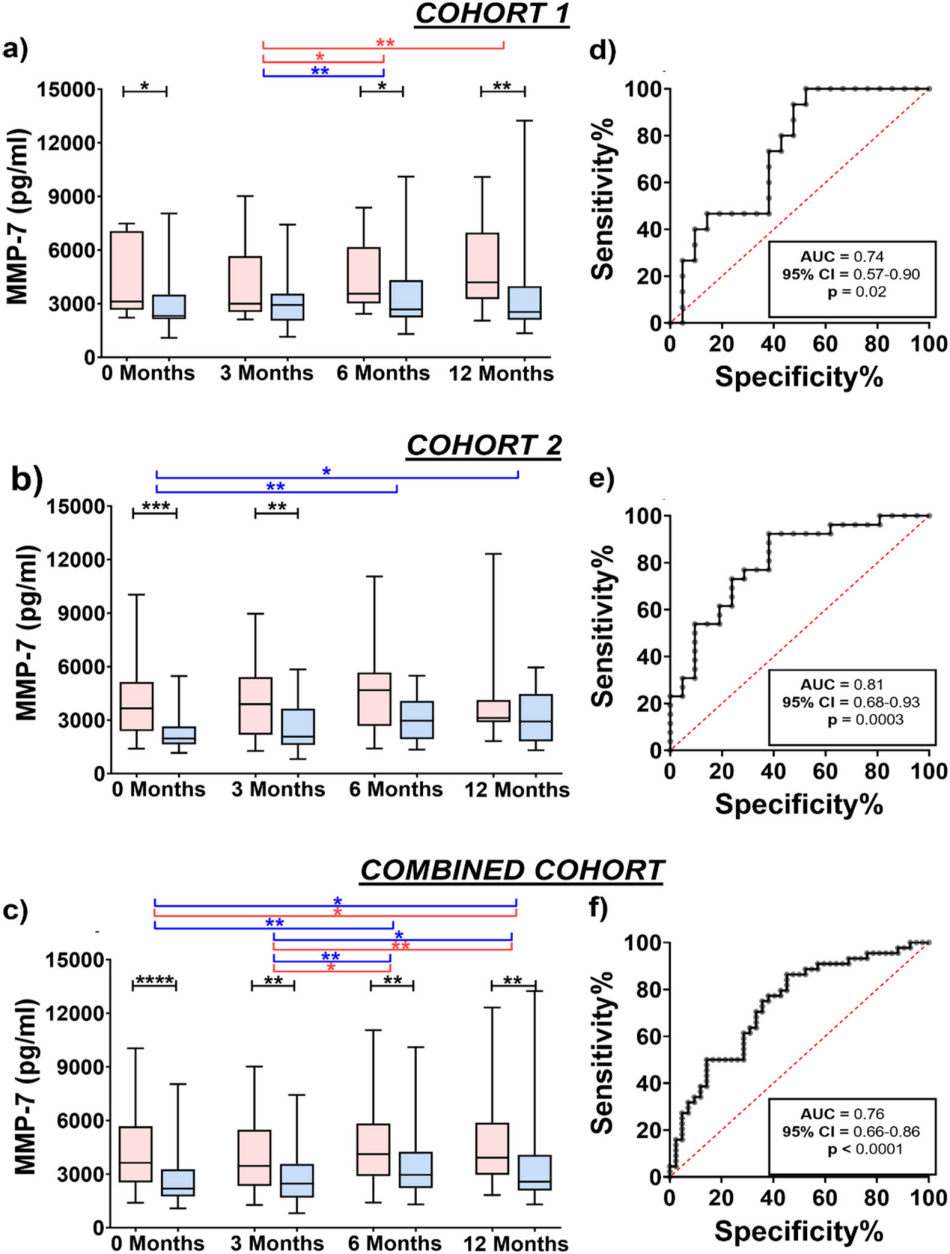
Progressive IPF patients is characterised by elevated levels of MMP7. Serum MMP7 levels in progressive (*red*) and stable (*blue*) at 0, 3, 6 and 12 months post‐antifibrotic treatment in the (a) Cohort 1, (b) Cohort 2 and (c) Combined cohort. Baseline AUC analysis of MMP7 for progression in the (d) Cohort 1, (e) Cohort 2 and (f) Combined cohort. Box represents interquartile range with median and whiskers represent range. *p < 0.05, **p < 0.01, ***p < 0.001, ****p < 0.0001.

Of the 10 molecules investigated, only baseline serum MMP7 level was higher in the progressive group compared to the stable group in both Cohort 1 (*p* = 0.02) and Cohort 2 (*p* = 0.0002) (Figure 1). In Cohort 1, MMP7 was also higher in progressive patients at 6 and 12 months (*p* = 0.03 and 0.004, respectively; Figure 1a). In Cohort 2, MMP7 was higher in progressive patients than stable patients at 3 months (*p* = 0.007; Figure 1b). Combined cohort analysis demonstrated that the levels of MMP7 were higher in progressive compared to stable patients at all timepoints (*p* < 0.01; Figure 1c).

Baseline MMP7 was the only marker that significantly differentiated progressive from stable patients in both Cohort 1 (AUC = 0.74, 95% CI = 0.57–0.90, *p* = 0.02; Figure 1d) and Cohort 2 (AUC = 0.81, 95% CI = 0.68–0.93, *p* = 0.0003; Figure 1e). The combined cohort analysis showed similar results (AUC = 0.76, 95% CI = 0.66–0.86, *p* < 0.0001; Figure 1f). ROC analysis showed that a threshold MMP7 value of 2.207 ng/mL had the best Youden index (maximum potential effectiveness) whereas MMP7 level of 2.414 ng/mL had a sensitivity of > 80% (Table 3).

**TABLE 3:** Categorical logistic regression analysis using MMP7 thresholds adjusted for gender, age and %pFVC.

MMP7 threshold (ng/mL)	Reason	Sensitivity (%)	Specificity (%)	Odds ratio	95% CI	*p* value
> 2.207	Highest Youden's index	90.2	54.8	9.253	2.806–37.92	0.001
> 2.414	Sensitivity > 80%	80.5	61.9	6.751	2.304–22.50	0.001
> 2.727	%Sen = %Spe	65.9	66.7	3.352	1.262–9.344	0.017
> 3.460	Specificity > 80%	51.2	81.0	3.659	1.313–10.91	0.015

Abbreviations: CI, confidence interval; MMP7, matrix metalloproteinase‐7.

**FIGURE 2:**
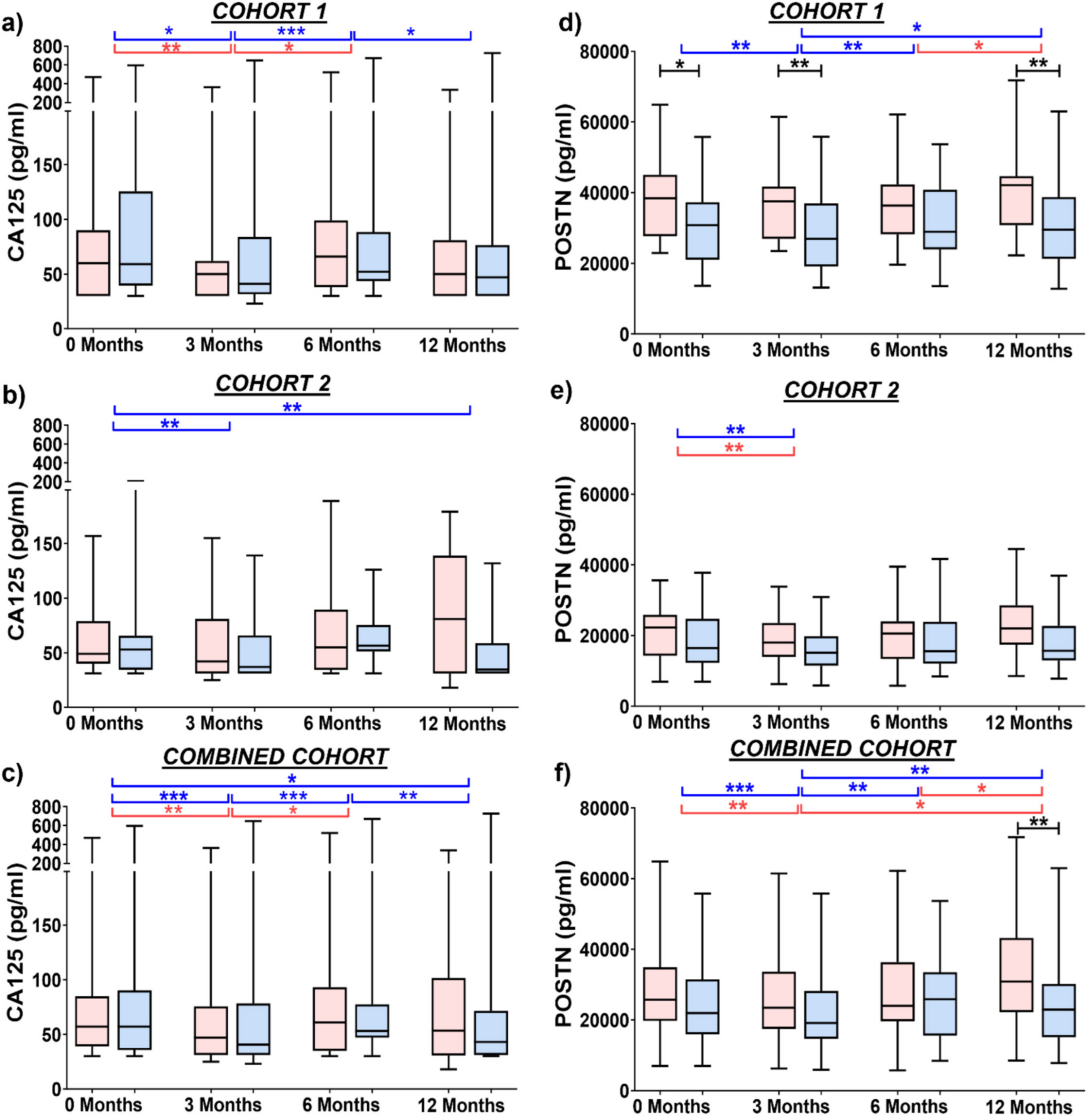
IPF stables showed decrease in CA125 and POSTN levels 3 months post‐antifibrotic treatment. Serum CA125 and POSTN levels in progressive (*red*) and stable (*blue*) at 0, 3, 6 and 12 months post‐antifibrotic treatment in the (a and d) Cohort 1, (b and e) Cohort 2 and (c and f) Combined cohort. Box represents interquartile range with median and whiskers represent range. *p < 0.05, **p < 0.01, ***p < 0.001.

Notably, serum CA125 levels decreased in stable patients from 0 to 3 months in both Cohort 1 (*p* = 0.03; Figure 2a) and Cohort 2 (*p* = 0.003; Figure 2b). Changes between other timepoints were observed but were not replicated. In a combined cohort analysis (Figure 2c), CA125 levels first decreased in both progressive and stable patients from 0 to 3 months but then increased from 3 to 6 months (*p* < 0.01). However, only stable patients showed a decrease in serum CA125 levels from 6 to 12 months (*p* = 0.003) and the levels at 12 months was lower compared to baseline (*p* = 0.02).

Serum POSTN levels also decreased in stable patients from 0 to 3 months in both Cohort 1 (*p* = 0.01; Figure 2d) and Cohort 2 (*p* = 0.007; Figure 2e). In a combined cohort analysis (Figure 2f), POSTN levels first decreased in both progressive and stable patients from 0 to 3 months. POSTN levels in stable patients then increased from 3 to 6 months (*p* = 0.002) and remained high at 12 months compared to 3 months (*p* = 0.003). Interestingly, POSTN level in progressive patients increased from 6 to 12 months (*p* = 0.02) above the levels in stable patients at 12 months (*p* = 0.01).

No other differences were replicated in both cohorts in the serum levels of ICAM‐1, OPN, SP‐D CXCL13, CCL18 and CHI3L1 (Supporting Information Figure S2).

### 3.3 Baseline Serum MMP7 Is Associated With 12‐Month Progression and Overall Mortality in IPF

Multivariate logistic regression analysis with all clinical and biomarker parameters together also showed that MMP7 was the only marker that is associated with progression (OR = 1.902, 95% CI = 1.247–3.162, *p* = 0.006; Table 4). With univariable logistic regression, gender, age, MMP7 and OPN were associated with progression (Supporting Information Table S2). After adjustment for gender, age and baseline %pFVC, only MMP7 was associated with 12‐month progression (odds ratio [OR] = 1.530, 95% CI = 1.145–2.199, *p* = 0.01), but not OPN. As expected, categorical analysis using MMP7 thresholds increased the OR (e.g., MMP7 of > 2.207 ng/mL: OR = 9.253, 95% CI = 2.806–37.92, *p* = 0.001; Table 3).

**TABLE 4:** Association of all baseline clinical parameters and biomarker levels with progression using a multiple logistic regression.

Association with progression			
Parameters	Odds ratio	95% CI	*p* value
Gender	0.346	0.075–1.472	0.157
Age	1.020	0.928–1.127	0.685
%pFVC	1.005	0.969–1.044	0.780
%pDLCO	1.005	0.963–1.049	0.829
MMP7 (ng/mL)	**1.902**	**1.247–3.162**	**0.006**
POSTN (ng/mL)	0.985	0.929–1.042	0.597
ICAM‐1 (ng/mL)	1.000	0.999–1.001	0.646
CXCL13 (pg/mL)	1.000	0.999–1.002	0.640
OPN (ng/mL)	1.008	0.978–1.04	0.606
SP‐D (ng/mL)	1.009	0.991–1.028	0.343
CHI3L1 (ng/mL)	1.003	0.998–1.008	0.245
CCL18 (ng/mL)	0.999	0.997–1.001	0.451
CA125 (pg/mL)	0.993	0.983–1.002	0.114

*Note*: Bold values highlight statistically significant results.

Abbreviations: CI, confidence interval; CA125, cancer antigen‐125 (mucin 16); CCL18, C‐C motif ligand 18; CHI3L1, chitinase‐3‐like protein‐1; CXCL13, C‐X‐C motif ligand 13; DLCO, diffusing capacity of the lungs for carbon monoxide; FVC, forced vital capacity; ICAM‐1, intercellular adhesion molecule‐1; MMP7, matrix metalloproteinase‐7; OPN, osteopontin; POSTN, periostin; SP‐D, surfactant protein‐D.

Overall mortality (time‐to‐event) was assessed in 98 patients of which 39 died during the follow up period (up to 52.7 months). Multivariate Cox regression with all clinical and biomarker parameters showed age, baseline %pFVC, MMP7 and CHI3L1 were associated with increased overall mortality risk with a c‐index of 0.74 (Supporting Information Table S4). Univariable Cox regression showed age, %pDLCO, GAP, MMP7, ICAM‐1 and CHI3L1 were associated with increased mortality risk (Supporting Information Table S3). After adjustment for gender, age and baseline %pFVC, MMP7 and CHI3L1 were associated with overall mortality (hazard ratio [HR] = 1.268, 95% CI = 1.086–1.464, *p* = 0.002 and HR = 1.003, 95% CI = 1.000–1.006, *p* = 0.018, respectively), ICAM‐1 was not. Categorical analysis using MMP7 thresholds (Table 3), showed that a threshold of > 3.46 ng/mL MMP7 was associated with a significant HR of 2.263 (95% CI = 1.146–4.517, *p* = 0.02) for overall mortality.

### 3.4 Biomarker‐Clinical Parameter Panel Predicts 12‐Month Progression and 3‐Year Mortality

LASSO regression was performed to produce predictive models using biomarkers (MMP7, CA125, POSTN, ICAM‐1, OPN, SP‐D, CXCL13, CCL18 and CHI3L1) with or without GAP score. Due to limited sample size, the cohorts were first analysed as a combined group. LASSO regression identified a biomarker panel consisting of MMP7, ICAM‐1, CHI3L1 and CA125 as the best predictor of progression (AUC = 0.78, *p* < 0.0001) and more discriminatory than MMP7 alone (AUC = 0.74, *p* = 0.0001; Table 5). Furthermore, the biomarker panel was predictive in both cohort 1 and 2 (Supporting Information Table S4). However, the biomarker panel was more discriminatory in Cohort 2 (AUC = 0.81, *p* = 0.0003) compared to Cohort 1 (AUC = 0.74, *p* = 0.01) (Supporting Information Table S4). GAP was not selected by LASSO as a predictive marker of progression in a combined cohort nor in separate cohorts (Table 5 and Supporting Information Table S4).

**TABLE 5:** AUC analysis of predictive progression and 3‐year mortality using parameters selected by LASSO regression.

Scoring system	Parameters selected by LASSO	AUC	*p*
Progression			
GAP	—	0.54	0.50
MMP7 only^a^	—	0.74	0.0001
Biomarker	MMP7, ICAM‐1, CHI3L1, CA125	0.78	< 0.0001
Biomarker + GAP^b^	MMP7, ICAM‐1, CHI3L1, CA125 GAP was not included as a predictive parameter by LASSO	NA	NA
3‐year mortality			
GAP	—	0.71	0.004
MMP7 only^a^	—	0.67	0.02
Biomarker	MMP7, ICAM‐1, SP‐D, CCL18	0.85	< 0.0001
Biomarker + GAP	MMP7, ICAM‐1, CCL18, GAP	0.87	< 0.0001

Abbreviations: AUC, area under curve; CA125, cancer antigen‐125 (mucin 16); CHI3L1, chitinase‐3‐like protein‐1; ICAM‐1, intercellular adhesion molecule‐1; LASSO, least absolute shrinkage and selection operator; MMP7, matrix metalloproteinase‐7; POSTN, periostin.

^a^ MMP7 AUC was analysed as an overall cohort.

^b^ GAP score was not selected by LASSO when included with biomarkers. Hence AUC and *p*‐values not available (NA).

**FIGURE 3:**
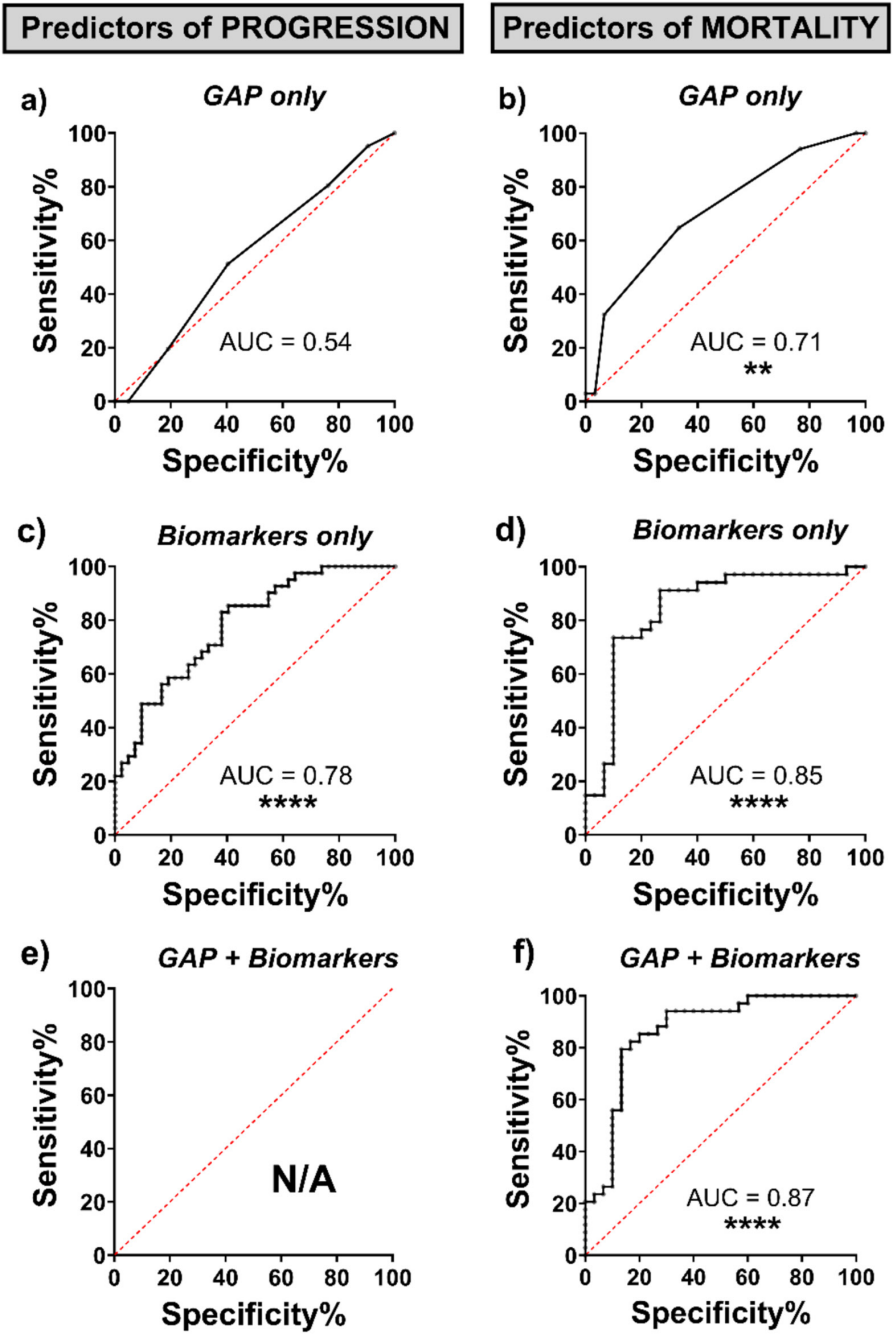
AUC analysis of GAP score in addition to biomarkers selected by least absolute shrinkage and selection operator (LASSO) regression models of progression and 3‐year mortality. The sensitivity and specificity were assessed via AUC of ROC analysis by comparing the predicted progression/mortality vs. actual progression/mortality using (a, b) GAP score only, (c, d) biomarkers only or (e, f) combined GAP and biomarkers. LASSO was applied to (c, d) biomarkers only and (e, f) combined GAP and biomarkers. (e) LASSO for combined GAP and biomarkers for progression did not include GAP as a predictor. Imputation was performed using classification and regression trees. **p < 0.01, ****p < 0.0001.

For 3‐year mortality, another biomarker panel consisting of MMP7, ICAM‐1, SP‐D and CCL18 was identified as more predictive (AUC = 0.85, *p* < 0.0001) than MMP7 (AUC = 0.67, *p* = 0.02) or GAP alone (AUC = 0.71, *p* = 0.004). However, combining these biomarkers with GAP improved the predictability of 3‐year mortality (AUC = 0.87, *p* < 0.0001) (Figure 3; Table 5). MMP7 combined with GAP score (AUC = 0.78, *p* = 0.0001) (Supporting Information Figure S4) was also analysed with an improvement over MMP7 alone but was outperformed by the biomarker panel both with and without GAP score.

The biomarker panels for 3‐year mortality were significantly replicated when separated into Cohort 1 (*n* = 39) and Cohort 2 (*n* = 25). However, the predictability of GAP score and MMP7 were not replicated. The biomarker panel without GAP (Cohort 1: AUC = 0.86, *p* < 0.0001 and Cohort 2: AUC = 0.68, *p* < 0.03) and with GAP (Cohort 1: AUC = 0.91, *p* < 0.0001 and Cohort 2: AUC = 0.73, *p* < 0.006) was significantly replicated in both cohorts although Cohort 2 had a small sample size (Alive *n* = 7, Deceased *n* = 17) (Supporting Information Table S4).

## 4 Discussion

In this study, we demonstrate that higher MMP7 levels pre‐treatment was associated with 12‐month progression and increased risk of mortality. Furthermore, although both progressive and stable patients showed an increase in MMP7 level at 12 months, MMP7 levels in progressive patients were higher than stable patients at all timepoints. Additionally, we demonstrate that categorical levels of MMP7 are associated with progression and mortality, thus bringing this biomarker closer to clinical use.

Our findings are in line with previous work which showed an inverse longitudinal relationship between MMP7 and FVC [16, 17], demonstrating the potential of MMP7 as a predictor of decline in IPF. Furthermore, circulating MMP7 is predictive of progression in untreated IPF patients as both a single marker [17] and part of a multi‐biomarker score [10]. However, these studies were in treatment‐naïve cohorts largely lacking longitudinal data. In antifibrotic‐treated IPF patients, Adegunsoye et al. found that higher MMP7 was predictive of worse survival and utilised MMP7 in a multi‐biomarker signature for mortality risk [12].

Interestingly our study showed increasing levels of MMP7 over 1‐year post‐treatment in both progressive and stable groups, with the MMP7 levels in progressive patients being higher than in stable patients at all timepoints. MMP7 is implicated in the pathogenesis of pulmonary fibrosis in human and animal studies. MMP7 is released by hypertrophic alveolar epithelial cells (AEC) with impaired regenerative ability [18]. MMP7 knockout mice have shown resistance to bleomycin induced pulmonary fibrosis [19]. Pro‐fibrotic molecules such as fibroblast growth factor (FGF), platelet derived growth factor (PDGF) and vascular endothelial growth factor (VEGF) induce the production of MMP7 through the Wnt/β‐catenin pathway in addition to the mitogen‐activate protein kinase (MAPK) and Akt signalling pathways responsible for fibroblast proliferation [20]. The increased MMP7 secretion by AECs may represent the elevated burden of impaired epithelial regeneration in IPF. MMP7 is also associated with extracellular matrix (ECM) remodelling and increased levels may reflect increased tissue remodelling and fibrosis in IPF [21].

As a tyrosine kinase inhibitor, nintedanib competitively binds FGF, PDGF and VEGF receptors thereby inhibiting pathways leading to MMP7 production [22] and fibroblast proliferation [23]. The Wnt/β‐catenin pathway is also activated by TGF‐β1 [24], a major mediator of fibrosis [25, 26]. Pirfenidone suppresses TGF‐β1 and so indirectly targets MMP7 production. The increasing level of MMP7 over 1‐year post‐antifibrotic treatment may reflect underlying progression despite anti‐fibrotic treatment, with disease activity reduced in stable IPF.

Baseline MMP7 was the only biomarker associated with 12‐month progression and baseline MMP7 and CHI3L1 with increased risk of mortality in our treated IPF cohort. Our data also supports the current literature that places GAP score as a marker of mortality in IPF [3, 27, 28]. Elevated MMP7, ICAM‐1 and CHI3L1 have been previously associated with increased mortality risk [12, 17, 29]. Notably, using LASSO regression, we identified MMP7, ICAM‐1 and CCL18 combined with GAP score best predicted 3‐year mortality. Interestingly, MMP7 and ICAM‐1 were utilised by previous studies that also developed multi‐parameter prognostic scores [10, 12].

Interestingly, there was a decrease in CA125 and POSTN levels in stable but not progressive patients after 3 months of antifibrotic treatment. In a combined cohort, there was a decrease in CA125 in stable IPF patients from 6 to 12 months that dropped below baseline levels, whilst POSTN increased in progressive patients at 12 months, above the level of the stable group. A previous study by Maher et al. reported that serum CA125 is elevated in progressive IPF at time of diagnosis and 3‐months post‐diagnosis in treatment‐naïve patients, and observed increased expression in IPF alveolar epithelium compared to healthy controls [11]. As an epithelial cell surface marker [30], elevated CA125 in serum may reflect the compromised barrier between basement alveolar epithelium and the bloodstream. POSTN is a matricellular protein typically expressed in the fibroblastic foci of lungs from IPF patients and bleomycin murine models [31]. We and others have also shown levels of circulatory POSTN to be predictive of lung function decline and mortality in IPF [10, 31].

Interestingly, we found CCL18, and SP‐D were predictive of 3‐year mortality while ICAM‐1, and CHI3L1 were predictive of overall mortality. Elevated serum CCL18 has previously been correlated with 24‐month mortality, similar to our findings for 3‐year mortality [32]. However, we found serum CCL18 levels did not correlate with overall mortality which included mortality further than 3‐years. Macrophages and monocytes are producers of CCL18, a T‐cell attractant. Elevated blood monocyte counts have been associated with elevated mortality risk in IPF patients [33]. Elevated SP‐D has been associated with increased overall mortality risk [34] but not at 1‐year mortality risk [12]. Our findings have shown both CCL18 and SP‐D are predictors of 3‐year mortality but not overall mortality (analysed by time‐to‐event), suggesting these molecules reflect the onset of decline in IPF.

The progressive patients in our cohort were older and had a higher proportion of females compared to the stable group but these demographic differences did not impact MMP7 levels. Although MMP7 increases with age, the levels measured in IPF are generally higher than in age matched non‐IPF and healthy individuals [16, 17, 35]. Our cohort was also limited by size and limited to Australian patients only. An additional cohort from another country or ethnicity would address both these issues. This would also validate our observations in an international group of patients. Additionally, cause of death was not recorded so deceased patients included in mortality analyses could not be confirmed to have passed due to IPF related reasons.

Despite current antifibrotics, patients with elevated MMP7 have increased risk of progression. Other studies of biomarkers to guide therapy have not demonstrated utility, as in the INMARK study [36], and further research is needed to determine the clinical application of MMP7. Our study supports the use of baseline and serial serum MMP7 levels as a predictive marker of IPF progression in antifibrotic treated patients and identifies CA125 as a potential marker of stability. Larger studies are required to determine how MMP7 and CA125 can be used to assist with clinical decision making.

## Author Contributions

**Roger M. Li:** data curation (lead), formal analysis (lead), investigation (lead), methodology (lead), validation (lead), visualization (lead), writing – original draft (lead), writing – review and editing (equal). **Dino B. A. Tan:** data curation (supporting), formal analysis (supporting), investigation (supporting), methodology (supporting), project administration (equal), supervision (equal), validation (supporting), visualization (supporting), writing – original draft (supporting), writing – review and editing (equal). **Chantalia Tedja:** data curation (equal), methodology (equal), project administration (supporting), writing – review and editing (equal). **Wendy A. Cooper:** data curation (equal), writing – review and editing (equal). **Helen E. Jo:** data curation (equal), writing – review and editing (equal). **Christopher Grainge:** data curation (equal), formal analysis (supporting), funding acquisition (supporting), writing – review and editing (equal). **Ian N. Glaspole:** data curation (equal), formal analysis (supporting), funding acquisition (supporting), writing – review and editing (equal). **Nicole Goh:** data curation (equal), formal analysis (supporting), writing – review and editing (equal). **Samantha Ellis:** data curation (equal), writing – review and editing (equal). **Peter M. A. Hopkins:** data curation (equal), writing – review and editing (equal). **Christopher Zappala:** data curation (equal), writing – review and editing (equal). **Gregory J. Keir:** data curation (equal), writing – review and editing (equal). **Paul N. Reynolds:** data curation (equal), formal analysis (supporting), funding acquisition (supporting), writing – review and editing (equal). **Sally Chapman:** data curation (equal), writing – review and editing (equal). **E. Haydn Walters:** formal analysis (supporting), funding acquisition (supporting), writing – original draft (supporting), writing – review and editing (equal). **Darryl Knight:** formal analysis (supporting), funding acquisition (supporting), writing – review and editing (equal). **Svetlana Baltic:** formal analysis (equal), funding acquisition (supporting), writing – review and editing (equal). **HuiJun Chih:** formal analysis (equal), writing – review and editing (equal). **Tamera J. Corte:** conceptualization (supporting), data curation (equal), formal analysis (equal), funding acquisition (equal), writing – original draft (supporting), writing – review and editing (equal). **Yuben P. Moodley:** conceptualization (lead), data curation (equal), formal analysis (equal), funding acquisition (lead), investigation (lead), methodology (equal), project administration (lead), resources (lead), supervision (lead), validation (supporting), visualization (supporting), writing – original draft (equal), writing – review and editing (equal).

## Ethics Statement

This study was performed in accordance with the Declaration of Helsinki. The AIPFR has human research ethics approval from all participating centres; the Sydney Local Health District Ethics Review Committee (protocols X11‐0287 and HREC/11/RPAH/439) and South Metropolitan Health Service Human Research Ethics Committee (PRN758). All adult participants provided written informed consent to participate in this study.

## Conflicts of Interest

During the peer review process, Paul N. Reynolds is Co‐Editor‐in‐Chief of the journal and co‐author of this article. He was excluded from the peer‐review process and all editorial decisions related to the acceptance and publication of this article. Peer‐review was handled independently by past Co‐Editor in Chief Philip G. Bardin and present Co‐Editor in Chief Toshiaki Kikuchi to minimise bias. In addition, Christopher Grainge, Darryl Knight, Tamera J Corte and Yuben P Moodley and were Editorial Board members of Respirology and co‐authors of this article. They were excluded from all editorial decision‐making related to the acceptance of this article for publication.

R. M. Li, D. B. A. Tan, C. Tedja, W. A. Cooper, S. Ellis, P. M. A. Hopkins, C. Zappala, G. J. Keir, P. N. Reynolds, E.H. Walters, H. J. Chih, D. Knight and S. Baltic has nothing to disclose. Y. P. Moodley, C. Grainge, I. N. Glaspole and S. Chapman reports personal fees for advisory board work from Boehringer Ingelheim and Roche, outside the submitted work. H. E. Jo reports other (travel support and lecture fees) from Boehringer Ingelheim and Roche, outside the submitted work. N. S. Goh reports grants from the National Health Medical Research Council (NHMRC) (APP1066128, APP114776) and the Centre of Research Excellence in Pulmonary Fibrosis (CRE‐PF), Australia (NHMRC GNT116371; 2017–2021), during the conduct of the study. T. J. Corte reports grants, personal fees and nonfinancial support from Boehringer Ingelheim, grants and personal fees from Roche, grants from Galapagos, Actelion and Bayer, outside the submitted work.

## Supporting information


Data S1.

Data S2.

## Data Availability

The data that support the findings of this study are available from the corresponding author upon reasonable request.
