# Supplementary material for: Pre‐Treatment MMP7 Predicts Progressive Idiopathic Pulmonary Fibrosis in Antifibrotic Treated Patients
Source: Respirology. 2025 Feb 7;30(6):504–14. doi: 10.1111/resp.14894 (PMC12128705; doi:10.1111/resp.14894)
Supplement: Supplementary file 1 — Data S1. [file RESP-30-504-s002.docx]

**SUPPORTING FIGURES**


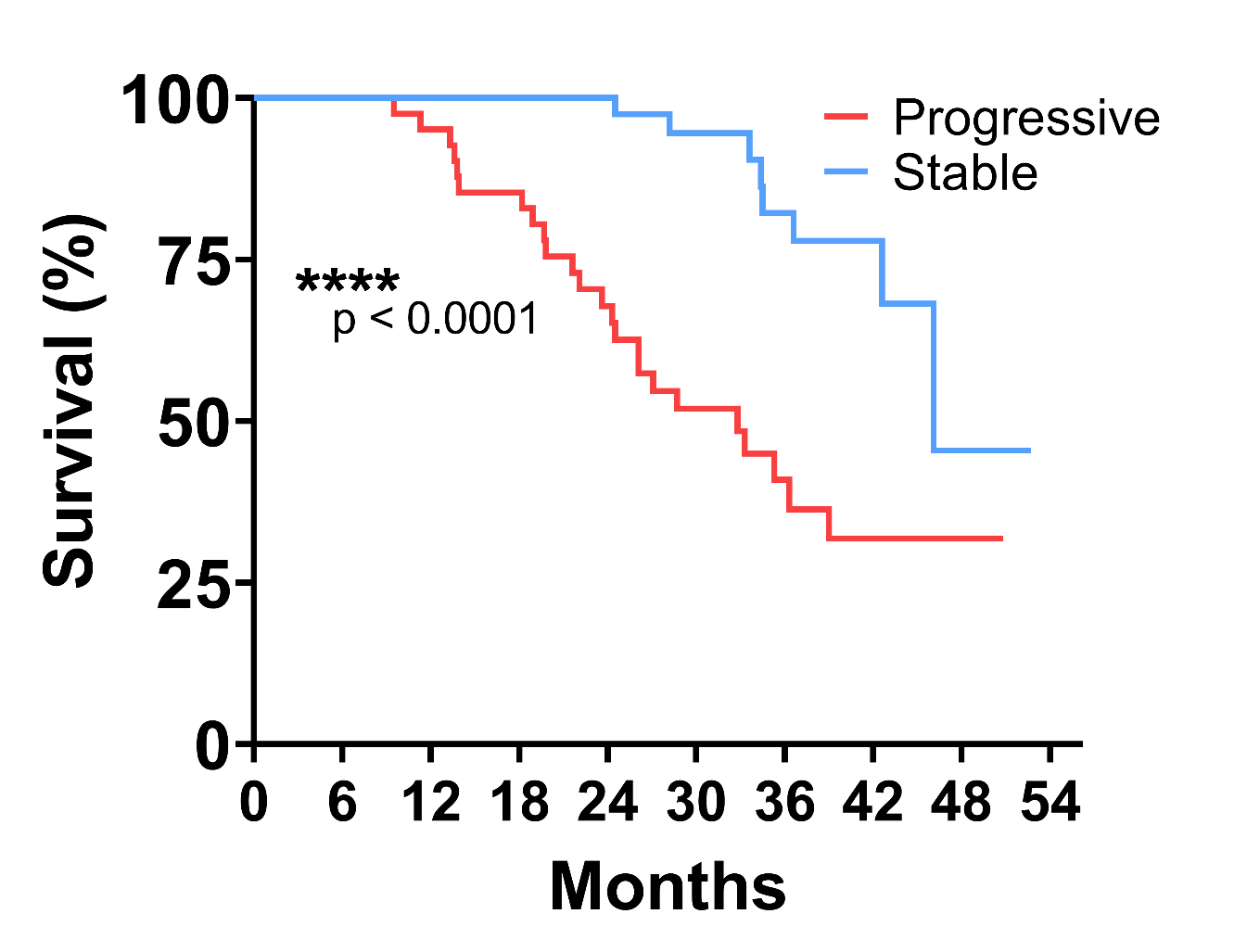


**Figure S1: Kaplan-Meier survival analysis of antifibrotic treated IPF patients based on lung function decline over 12-months after start of treatment.** Progressive patients *(red)* had an overall higher risk of mortality than stable patients *(blue)*.

**
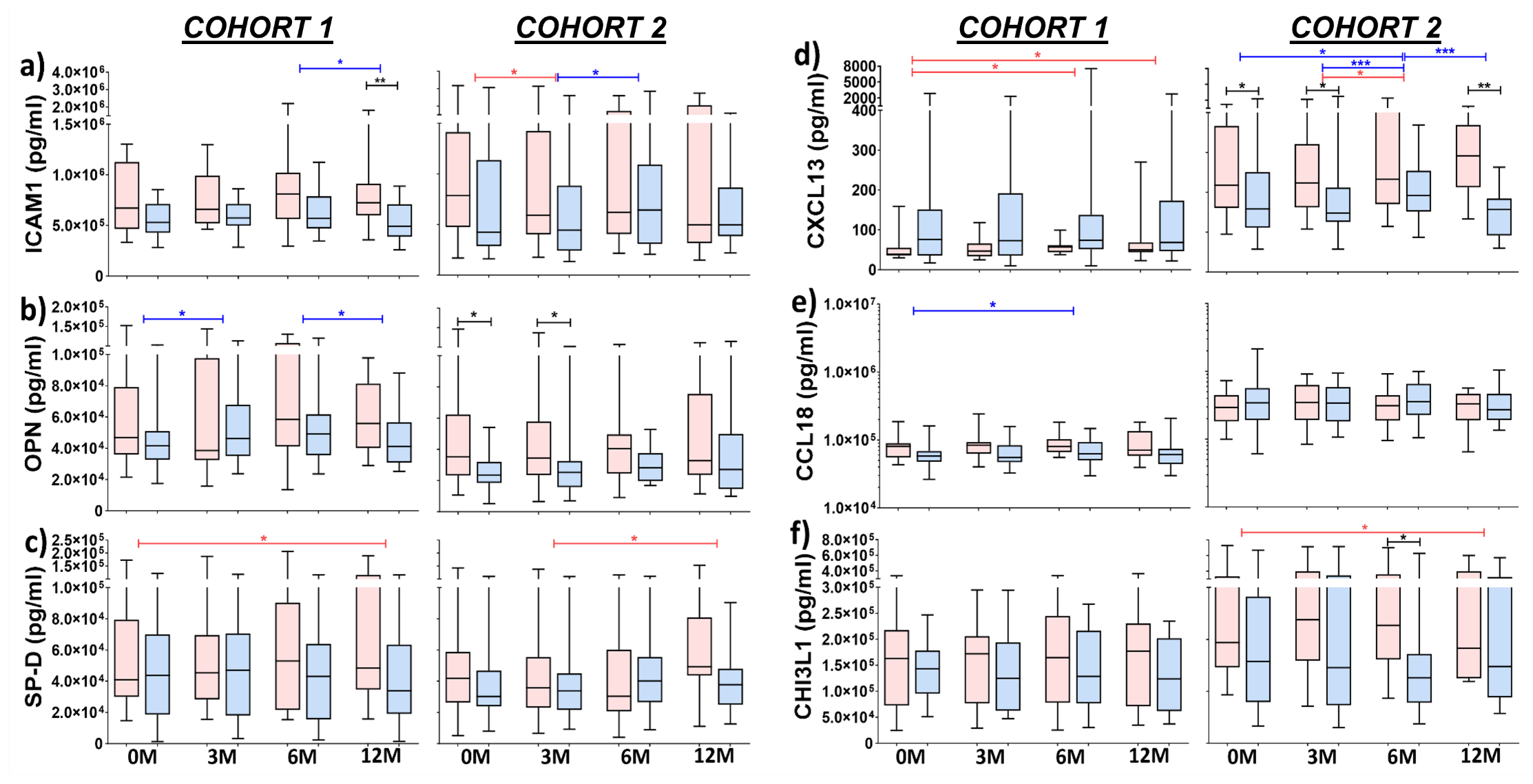
 Figure S2: Serum concentrations of (a) ICAM-1, (b) OPN, (c) SP-D, (d) CXCL13, (e) CCL18 and (f) CHI3L1 in progressive *(red)* and stable *(blue)* at 0, 3, 6 and 12 months post-antifibrotic treatment in Cohort 1 *(left panel)* and Cohort 2 *(right panel)*.** *CCL18 displayed on a log scale due to the spread of concentrations. Box represents interquartile range with median and whiskers represent range.
*p < 0.05, **p < 0.01, ***p < 0.001*


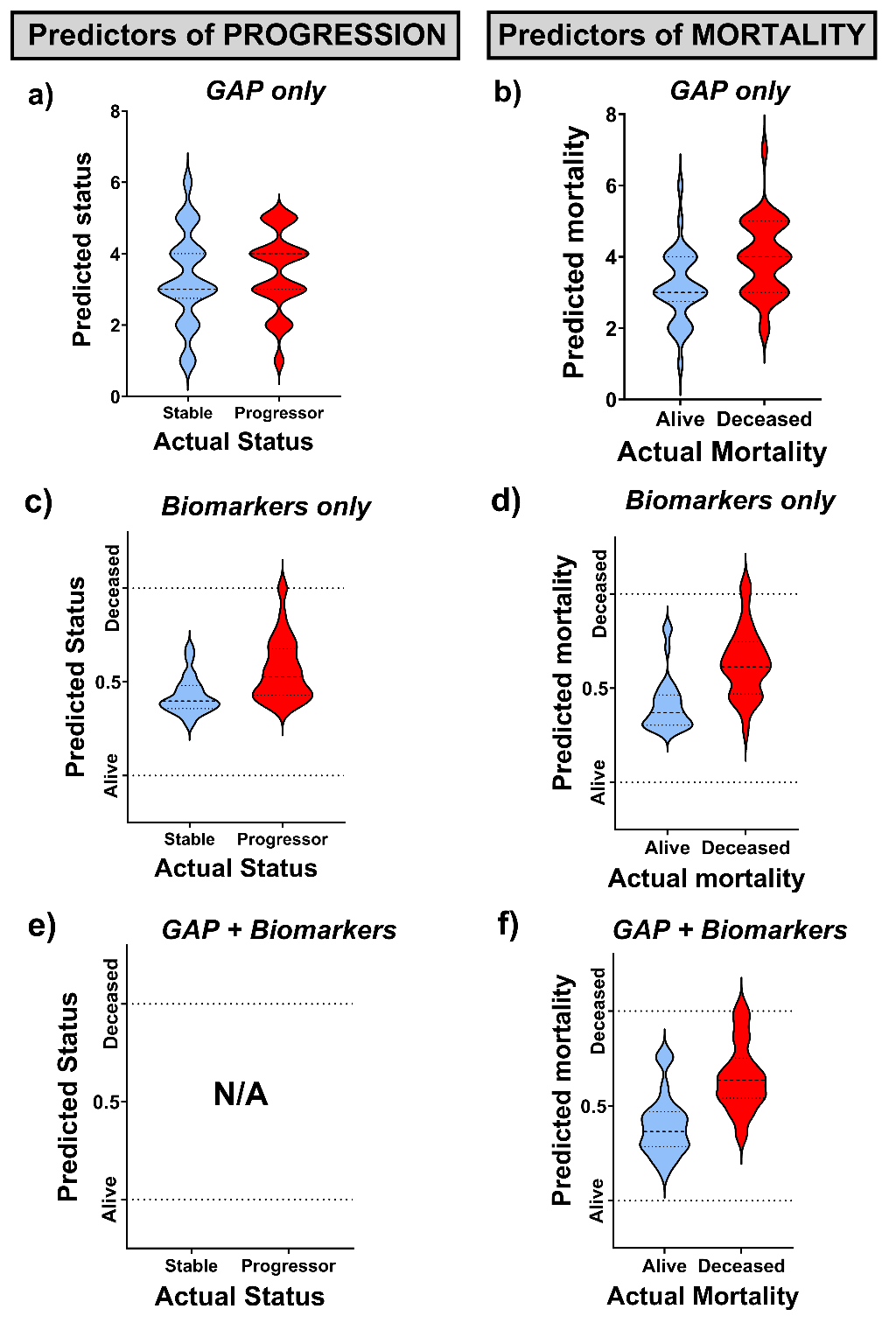


**Figure S3: Comparison of GAP score in addition to biomarkers selected by least absolute shrinkage and selection operator (LASSO) regression models of progression and 3-year mortality.** The spread and comparison of predicted progression/mortality vs actual progression/mortality using **(a-b)** GAP score, **(c-d)** biomarker values and **(e-f)** combined GAP score and biomarkers values. LASSO was applied to **(c-d)** biomarkers only and **(e-f)** combined GAP and biomarkers. **(e)** LASSO for combined GAP and biomarkers for progression did not include GAP as a predictor. Progressive and deceased patients in red and stable and alive patients in blue. *Imputation was performed using classification and regression trees.*


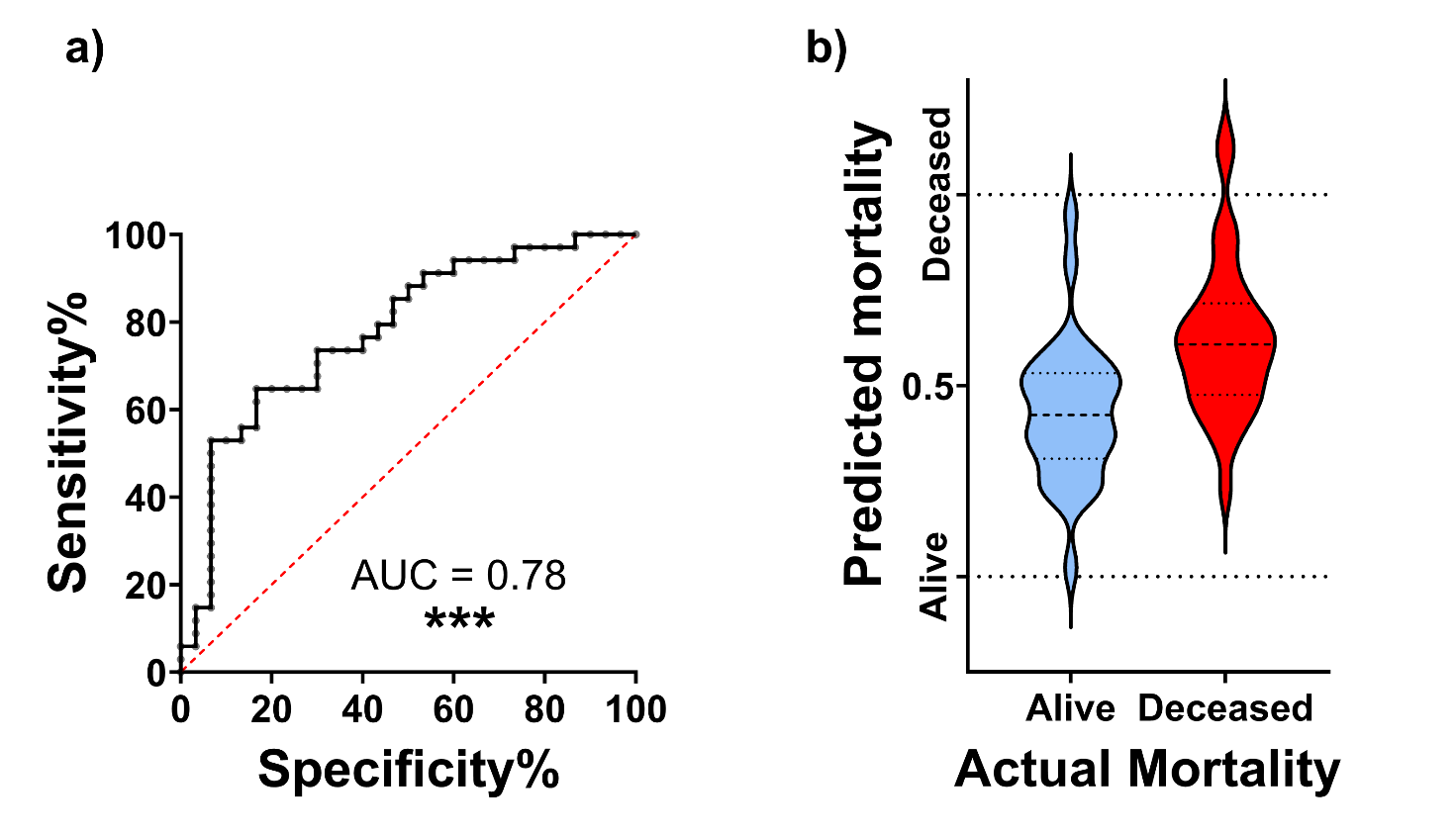


**Figure S4: AUC analysis of a least absolute shrinkage and selection operator (LASSO) regression model of MMP7 and GAP score for 3-year mortality. a)** The sensitivity and specificity were assessed via AUC of ROC analysis by comparing the predicted mortality vs actual mortality. **b)** The difference between the spread and comparison of predicted vs actual mortality. The LASSO model ignored GAP score when assessing progression and was not included. *Imputation was performed using classification and regression trees. *** p<0.001*
