# Supplementary material for: Pre‐Treatment MMP7 Predicts Progressive Idiopathic Pulmonary Fibrosis in Antifibrotic Treated Patients
Source: Respirology. 2025 Feb 7;30(6):504–14. doi: 10.1111/resp.14894 (PMC12128705; doi:10.1111/resp.14894)
Supplement: Supplementary file 2 — Data S2. [file RESP-30-504-s001.docx]

**SUPPORTING TABLES**

**Table S1: Patient characteristics in Cohort 1 and Cohort 2**

| **Characteristics** | **Cohort 1**  **(n = 43)** | **Cohort 2**  **(n = 55)** | **p-value** |
| --- | --- | --- | --- |
| Progressive^1^ | 15 | 26 | 0.20 |
| Stable | 21 | 21 |  |
| Unknown^2^ | 7 | 8 |  |
| Age | 75 (69-79) | 73 (67-76) | 0.11 |
| Male | 29 | 46 | 0.09 |
| Female | 14 | 10 |  |
| Caucasian | 36 | 52 | 0.15 |
| Not Caucasian | 7 | 4 |  |
| BMI | 28 (26-29) | 29 (26-32) | 0.08 |
| %predicted FVC baseline | 94 (74-104) | 85 (77-97) | 0.18 |
| %predicted DLCO baseline | 52 (39-63) | 54 (43-67) | 0.27 |
| GAP Score (mean ± SD) | 3.56 ± 1.24 | 3.38 ± 1.2 | 0.69 |
| Nintedanib | 16 | 30 | 0.24 |
| Pirfenidone | 18 | 19 |  |
| ^3^Nintedanib and Pirfenidone | 9 | 7 |  |
| Ever smoked | 25 | 40 | 0.17 |
| Never Smoked | 17 | 15 |  |

*BMI, body mass index; FVC, forced vital capacity; DLCO, diffusing capacity of the lungs for carbon monoxide; GAP, gender, age and physiology; SD, standard deviation. Continuous variables are displayed as median and interquartile range (IQR) unless stated otherwise.*

*^1^Progressive IPF patients were identified as patients who experienced a ≥10% decline in FVC or ≥15% decline in DLCO over 6-12 months post-treatment or who were deceased within 12 months after their baseline blood sample.*

*^2^Patients with unknown progressive/stable status were still included in 3-year mortality analysis.*

*^3^Patients started on Nintedanib or Pirfenidone who then switched to the other during follow up.*

**Table S2: Association of individual baseline clinical parameters and biomarker levels with progression using a univariable simple logistic regression.**

| **Association with PROGRESSION** | | | |
| --- | --- | --- | --- |
| **Parameters** | **Odds ratio** | **95% CI** | **p-value** |
| **Gender (Male)** | **0.321** | **0.102-0.912** | **0.039** |
| **Age** | **1.092** | **1.024-1.175** | **0.011** |
| **%pFVC** | 1.020 | 0.994-1.048 | 0.141 |
| **%pDLCO** | 0.984 | 0.955-1.013 | 0.288 |
| **GAP** | 1.113 | 0.777-1.608 | 0.560 |
| **MMP7 (ng/mL)** | **1.656** | **1.249-2.343** | **0.002** |
| **POSTN (ng/mL)** | 1.022 | 0.985-1.062 | 0.257 |
| **ICAM-1 (µg/mL)** | 1.001 | 1.000-1.002 | 0.065 |
| **CXCL13 (pg/mL)** | 1.000 | 0.999-1.001 | 0.710 |
| **OPN (ng/mL)** | **1.022** | **1.003-1.044** | **0.035** |
| **SP-D (ng/mL)** | 1.008 | 0.994-1.023 | 0.273 |
| **CHI3L1 (ng/mL)** | 1.003 | 1.000-1.007 | 0.074 |
| **CCL18 (ng/mL)** | 1.000 | 0.998-1.001 | 0.776 |
| **CA125 (pg/mL)** | 0.998 | 0.991-1.003 | 0.480 |

*CI, Confidence interval; CA125, cancer antigen-125 (mucin 16); CCL18, C-C motif ligand 18; CHI3L1, chitinase-3-like protein-1; CXCL13, C-X-C motif ligand 13; DLCO, diffusing capacity of the lungs for carbon monoxide; FVC, forced vital capacity; ICAM-1, intercellular adhesion molecule-1; MMP7, matrix metalloproteinase-7; OPN, osteopontin; POSTN, periostin; SP-D, surfactant protein-D*

**Table S3: Association of individual baseline clinical parameters and biomarker levels with overall (time-to-event) mortality using a univariable Cox Hazard regression.**

|  | **Association with overall MORTALITY** | | | | |
| --- | --- | --- | --- | --- | --- |
| **Parameters** | | **Hazard ratio** | **95% CI** | **p-value** | **Harrell’s C-statistic** |
| **Gender (Male)** | | 0.921 | 0.468-1.94 | 0.819 | 0.49 |
| **Age** | | **1.053** | **1.010-1.098** | **0.016** | **0.64** |
| **%pFVC** | | 0.985 | 0.970-1.000 | 0.060 | 0.58 |
| **%pDLCO** | | **0.969** | **0.950-0.989** | **0.002** | **0.65** |
| **GAP** | | **1.576** | **1.217-2.025** | **0.0005** | **0.66** |
| **MMP7 (ng/mL)** | | **1.253** | **1.087-1.429** | **0.001** | **0.63** |
| **POSTN (ng/mL)** | | 1.014 | 0.985-1.042 | 0.333 | 0.50 |
| **ICAM-1 (µg/mL)** | | **1.695** | **1.064-2.549** | **0.017** | **0.65** |
| **CXCL13 (pg/mL)** | | 1.000 | 1.000-1.001 | 0.330 | 0.59 |
| **OPN (ng/mL)** | | 1.008 | 0.998-1.017 | 0.100 | 0.60 |
| **SP-D (ng/mL)** | | 1.004 | 0.994-1.012 | 0.412 | 0.57 |
| **CHI3L1 (ng/mL)** | | **1.003** | **1.001-1.005** | **0.007** | **0.65** |
| **CCL18 (ng/mL)** | | 1.001 | 0.999-1.001 | 0.320 | 0.63 |
| **CA125 (pg/mL)** | | 1.001 | 0.999-1.002 | 0.134 | 0.56 |

*CI, Confidence interval; CA125, cancer antigen-125 (mucin 16); CCL18, C-C motif ligand 18; CHI3L1, chitinase-3-like protein-1; CXCL13, C-X-C motif ligand 13; DLCO, diffusing capacity of the lungs for carbon monoxide; FVC, forced vital capacity; GAP, gender, age and physiology; ICAM-1, intercellular adhesion molecule-1; MMP7, matrix metalloproteinase-7; OPN, osteopontin; POSTN, periostin; SP-D, surfactant protein-D*

**Table S4: Cohort 1 and Cohort 2 AUC analysis of predictive progression and 3-year mortality using parameters selected by LASSO regression.**

| **Scoring system** | **AUC** | **p-value** |  | **AUC** | **p-value** |
| --- | --- | --- | --- | --- | --- |
|  | **Cohort 1** | | **Cohort 2** | | |
| ***PROGRESSION*** | | |  | | |
| **GAP** | 0.63 | 0.2 |  | 0.61 | 0.21 |
| **MMP7 only^1^** | **0.74** | **0.02** |  | **0.81** | **0.0003** |
| **Biomarker** | **0.74** | **0.01** |  | **0.79** | **0.0004** |
| **Biomarker + GAP^2^** | **NA** | **NA** |  | **NA** | **NA** |
| ***3-Year Mortality*** | | |  | | |
| **GAP** | **0.76** | **0.004** |  | 0.65 | 0.07 |
| **MMP7 only^3^** | **0.85** | **0.0003** |  | 0.59 | 0.50 |
| **Biomarker** | **0.86** | **<0.0001** |  | **0.68** | **0.03** |
| **Biomarker + GAP** | **0.91** | **<0.0001** |  | **0.73** | **0.006** |

*AUC, area under curve; CA125, cancer antigen-125 (mucin 16); CHI3L1, chitinase-3-like protein-1; ICAM-1, intercellular adhesion molecule-1; LASSO, least absolute shrinkage and selection operator; MMP7, matrix metalloproteinase-7; POSTN, periostin*

***^1^****MMP7 AUC was analysed as an overall cohort.*

***^2^****GAP score was not selected by LASSO when included with biomarkers. Hence AUC and p-values not available (NA)*

*^3^MMP7 mortality measured as AUC of 3-year mortality*
